# Promising Biotechnological Applications of the Artificial Derivatives Designed and Constructed from Plant microRNA Genes

**DOI:** 10.3390/plants14030325

**Published:** 2025-01-22

**Authors:** T. N. Erokhina, Ekaterina V. Ryabukhina, Irina S. Lyapina, Dmitry Y. Ryazantsev, Sergey K. Zavriev, Sergey Y. Morozov

**Affiliations:** 1 Shemyakin-Ovchinnikov Institute of Bioorganic Chemistry, Russian Academy of Sciences, 117997 Moscow, Russia; tne@mx.ibch.ru (T.N.E.); ek.ryabukhina@yandex.ru (E.V.R.); amadeynemez@gmail.com (I.S.L.); d.yu.ryazantsev@gmail.com (D.Y.R.); szavriev@ibch.ru (S.K.Z.); 2 Belozersky Institute of Physico-Chemical Biology and Biological Faculty, Lomonosov Moscow State University, 119991 Moscow, Russia

**Keywords:** microRNA, gene expression, gene regulation, amiRNA, miPEP, plant virus, plant biotechnology, plant pest organisms, biotic and abiotic stresses

## Abstract

MicroRNAs (miRNAs) are small regulatory RNAs that are expressed in a tissue-specific manner during the development of plants and animals. The genes of miRNAs have been found to produce the following two products: (i) primary transcripts of these genes (pri-miRNA) are processed to give rise to mature miRNA, and (ii) in some cases, the pri-miRNA molecules can be translated to form small peptides, named as miPEPs. Gene silencing by artificial microRNAs (amiRNAs) is one of the potential crucial methods for the regulation of desired genes to improve horticultural plants. Likewise, external application of chemically synthesized miPEPs may help plants to resist biotic/abiotic stresses and grow faster. These potent and reliable derivatives of miRNA genes can be applied for improving useful traits in crop plants. This review summarizes the progress in research on the artificial gene derivatives involved in regulating plant development, virus and pest diseases, and abiotic stress resistance pathways. We also briefly discuss the molecular mechanisms of relevant target genes for future research on breeding in plants. In general, this review may be useful to researchers who are implementing amiRNA and miPEP for accelerating breeding programs and developmental studies in crop plants.

## 1. Introduction

Genes of microRNAs (miRNAs) in plants and animals are transcribed in the nucleus from chromosomal DNA by RNA polymerase II. The primary miRNA transcripts, called pri-miRNAs, have been shown to contain 5′-terminal cap structure and 3′-terminal poly(A) tails [1,2,3,4]. Like promoters in protein-coding genes, the miRNA promoters consist of TATA box motifs (about 30 bases upstream of the transcription start site), CAAT box motifs (about 85 bases upstream of the transcription start site), and other cis-acting elements [5]. Transcription initiation on TATA-containing promoters requires the assembly of the preinitiation complex. This process is triggered by an interaction of TATA-binding protein (TBP), a component of the general transcription factor TFIID, with a TATA box. Existing data show that TATA boxes are involved not only in the initiation of transcription machinery assembly but also in plant adaptation to environmental conditions, responding to light and other phenomena. The other numerous cis-acting elements include, particularly, elements regulating plant growth and development, hormone response, and stress response [3,4,5]. The pri-miRNAs have been shown to include different numbers of specific internal imperfect hairpins (single, two, or several), which are designated as pre-miRNAs [1,2,3,4]. Plant pre-miRNAs are variable in length (from 60 nts to over 500 nts) and are capable of highly specific nuclease processing by a processor complex. This complex includes an RNAse III-type endoribonuclease, DCL1, which first cuts at the loop-distal site of the hairpin, and the second processing step is cutting off the loop [1,2,3,4]. The resulting short, imperfect double-stranded RNAs (usually 21–24 bp) having 3′-extending two-nucleotide overhangs and undergoing subsequent 3′-terminal methylation by the RNA methyltransferase HEN1 represent mature miRNAs [3,4,6]. After transport from nucleus to cytoplasm, one strand of the duplex mature miRNA (the guide strand) is included into an ARGONAUTE (AGO) protein complex, resulting in an RNA-induced silencing complex (RISC). The AGO protein is characterized by four major structural domains: a N-terminal domain (NTE), a conserved PAZ domain, a conserved MID domain, and a conserved PIWI domain. The conformation of the folded AGO protein displays a bilobate structure. The NTE is necessary for loading miRNAs into AGO1, independent of its role in AGO1’s nuclear–cytoplasmic shuttling. The MID domain recognizes the 5′ termini of the miRNA, whereas the PAZ domain secures the 3′ termini. The PIWI domain is distinguished by its endonuclease activity. The guide strand, included in RISC, recognizes the target RNA transcripts, and RISC represses target gene expression through translational inhibition and/or RNA cleavage [1,3,4,6].

Many studies have revealed that the activity of plant miRNAs plays an important role in the flowering cycle, the absorption of nutrients, hormone action, the stress response, root initiation, leaf development, vascular development, flower development, phase transition, and seed development [7,8,9,10,11,12,13,14]. Evidently, assuming these data, miRNA-based approaches have great potential in biotechnological applications, particularly in triggering stress resistance of different crop species [15,16]. Indeed, human and animal miRNA studies have shown significant efficiency of chemically synthesized miRNA mimics and artificially designed miRNA in anti-cancer and anti-virus therapeutics [17,18,19,20,21,22,23,24]. The approach with exogenous chemically synthesized and externally sprayed miRNA duplexes has been shown to be also functional in plants and insects [25,26,27,28,29]. In the same time, genetic engineering approaches with pre-miRNA of artificial miRNA (amiRNA) have been found to enhance crop yield and stress resistance of plants, resulting in excellent agricultural potential [30,31,32,33,34,35]. Likewise, human and animal pre-amiRNAs are useful therapeutic tools for a broad spectrum of diseases, including neurodegenerative diseases, cancers, and viral infections [18,36,37].

In recent years, it has been shown that mature miRNA is not the only biologically active molecular product of miRNA genes. Many papers have reported that a significant number of plant and animal pri-miRNAs encode functional peptides (miPEPs) with a size of around 5 to 200 amino acids. These miPEPs can function as expression regulators for their corresponding pri-miRNAs and can influence plant growth and tolerance to stresses [38,39,40,41]. In this review, we mainly focus on some potential practical applications of plant amiRNA and miPEPs. The specific aim of this review is to summarize the physiological effects on crop yield enhancement and the biotic/abiotic stress protection activity of these miRNA gene derivatives, which are transgenically expressed in planta or exogenously applied to plants.

## 2. Potential Biotechnological Impact of amiRNA

**Figure 1:**
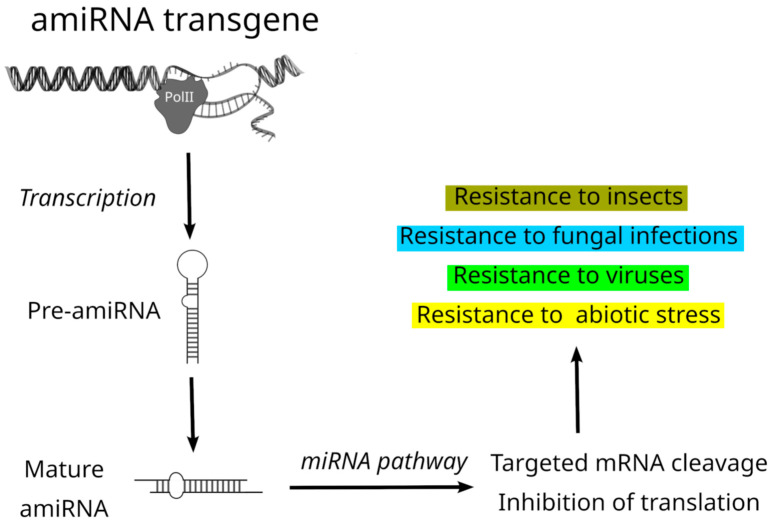
Schematic representation of the amiRNA pathway in transgenic plants to improve resistance to biotic and abiotic stresses.

Construction of amiRNA includes genetic modification of the miRNA gene to replace part of the pre-miRNA backbone, namely, the miRNA/miRNA* duplex with an artificially designed amiRNA/amiRNA* duplex. Thus, the amiRNA chain contains customized sequences to silence one or more genes of interest in various plant species by loading into the RISC, while the amiRNA* strand is expected to be degraded [31,42,43,44]. These species include mainly *Arabidopsis thaliana*, *Nicotiana benthamiana*, *Nicotiana tabacum*, *Solanum tuberosum*, *Oryza sativa*, *Triticum aestivum*, and *Cucumis sativus*. Backbones of different plant pre-miRNAs have been used to design amiRNAs, namely, those from miR172a, miR159b, miR164a, miR165a, miR166g, miR167a, miR319a, miR395a, miR390a, and miR528 [30,31,33,34,45,46]. Importantly, when both miRNA/miRNA* sequences are altered without changing structural features such as mismatches or bulges, this often leads to high-level accumulation of miRNA of the desired sequence. It has been demonstrated that not only reporter genes but also endogenous genes can be targeted with amiRNAs. These constructs seem to work with similar efficiency in different plant species mentioned above. Moreover, amiRNAs are effective when expressed from either constitutive or tissue-specific promoters. Most importantly, their sequences can be easily optimized to silence one or several target transcripts without affecting the expression of other transcripts. Early studies with plant amiRNA in *A. thaliana* have shown the functional efficiency of specific backbone replacement with sequences complementary to the GFP gene on the basis of pre-miR171 [47] and to endogenous plant genes using pre-miR165b [48] or pre-miR172a and pre-miR319a [42]. Design of amiRNA backbones, which are expressed transiently or in transgenes and targeting plant and foreign sequences, has also shown the ability of artificially designed miRNA constructs to target and inhibit the activity of viral messengers as well as plant and pathogen mRNAs influencing the survival of insects and fungi. Importantly, multiple amiRNAs can be inserted in a single gene expression cassette to develop transgenic plants resistant to several pathogens [27,28,30,32,34,35,42,46,49,50,51] (Figure 1).

### 2.1. Anti-Viral Activity of amiRNA Expressed Transiently and Through Stable Transgenesis

In this pioneering study, amiRNAs were used to confer single and dual resistance against two positive-stranded RNA viruses, Turnip yellow mosaic virus (TYMV, family Tymoviridae) and Turnip mosaic virus (TuMV, family Potyviridae), in transgenic *A. thaliana* plants expressing one or two amiRNA transgenes based on miR159a [50].

In later studies, amiRNAs were used against additional positive-stranded RNA viruses, namely, *Cucumber mosaic virus* (family Bromoviridae), *Odontoglossum ringspot virus* and *Cucumber green mottle mosaic* virus (family Virgaviridae), *Tomato bushy stunt virus* (family Tombusviridae), *Potato virus X* (family Alphaflexiviridae), and *Grapevine fanleaf virus* (family Secoviridae). Moreover, the amiRNA approach has been applied to double-stranded RNA viruses, such as the *Rice black streaked dwarf virus* (family Spinareoviridae) and minus-stranded RNA viruses, such as the *Rice stripe virus* (family Phenuiviridae) and *Watermelon silver mottle tospovirus* (family Tospoviridae), as well as to several geminiviruses (family Geminiviridae) and viroids (Potato spindle tuber viroid) (Table 1).

#### 2.1.1. Families Tymoviridae and Potyviridae

The above-mentioned amiRNAs were designed to target the genes of viral silencing suppressor proteins (VSRs), namely, P69 and HC-Pro of TYMV and TuMV [50]. Plant virus VSR proteins use multiple mechanisms to inhibit silencing. Many VSRs can compete with RISC by efficiently binding double-stranded small interfering RNAs. Additionally, the counter-defense role of some other VSRs is based on the degradation of AGO proteins via the proteasome pathway [52]. 

The data on TuMV have been confirmed by additional studies. Furthermore, these studies have demonstrated that combining more than one amiRNA into the same transgenic plant, targeting two or more virus genes, conferred more robust resistance. Additionally, the transgenic *A. thaliana* plants maintained resistance to TuMV when this virus was co-inoculated with other unrelated viruses [53,54]. In further studies, several other members of Potyviridae have also been shown to be inhibited in plants by the amiRNA method. Particularly, the amiRNA transgenes based on miR159a and targeting several genes in the ipomovirus genomes (Cassava brown streak disease—CBSV, and Ugandan cassava brown streak virus—UCBSV, family Potyviridae) gave rise to significant resistance in *N. benthamiana* plants. Moreover, two amiRNAs targeting P1 and NIb genes of CBSV showed protection against both CBSV and UCBSV [55]. Importantly, among amiRNAs targeting several Soybean mosaic virus (SMV) genes, P1 gene-targeting amiRNA has the highest antiviral activity in a transient amiRNA expression assay and in transgenic *N. benthamiana* [56] (Table 1).

Highly specific and effective resistance against Potato virus Y (PVY) has been shown using targeting of the potyvirus HC-Pro gene by amiRNAs based on miR159a, miR167b, and miR171a backbones in transgenic *N. tabacum* [57] and *S. tuberosum* [58] (Table 1). Importantly, both above studies have shown that transgenic plants developed high resistance to several unrelated viruses through the expression of multimeric amiRNA constructs, which included several pre-miRNA hairpins specific for PVY and Potato virus X. The ability of miR319a-based amiRNA targeting NIb gene to significantly inhibit replication of the PVY necrotic strain has been revealed in transgenic *N. tabacum* plants [59,60].

**Table 1:** Examples of the amiRNA-induced antiviral resistance in plants.

Target Virus Family and Name	miRNA Used as Backbone	Expression Method	Reference
***Tymoviridae*,** Turnip yellow mosaic virus *	miR159a	transgene	[50]
***Potyviridae*,** Turnip mosaic virus	miR159a	transgene	[50]
***Potyviridae*,** Turnip mosaic virus	miR159a	transgene	[53,54]
***Potyviridae*,** Cassava brown streak disease virus	miR159a	transgene	[55]
***Potyviridae*,** Ugandan cassava brown streak virus	miR159a	transgene	[55]
***Potyviridae*,** Soybean mosaic virus	miR159a	transient transgene	[56]
***Potyviridae*,** Potato virus Y	miR159a, miR167b, miR171a	transgene	[57]
***Potyviridae*,** Potato virus Y	miR159a	transgene	[58]
***Potyviridae*,** Potato virus Y (necrotic strain)	miR319a	transgene	[59,60]
***Potyviridae*,** Tobacco etch virus	miR319a	transgene	[59]
***Potyviridae*,** Wheat streak mosaic virus	miR395	transient transgene	[61]
***Potyviridae*,** Wheat yellow mosaic virus	os-miR528	transgene	[62]
***Potyviridae*,** Plum pox virus	miR159a	transgene	[63]
***Potyviridae*,** Zucchini yellow mosaic virus	miR390a	transient	[64]
***Bromoviridae***, Cucumber mosaic virus	miR171a	transgene	[65]
***Bromoviridae***, Cucumber mosaic virus	miR159a	transgene	[66]
***Bromoviridae***, Cucumber mosaic virus	miR171a	transgene	[67]
***Virgaviridae***, Odontoglossum ringspot virus	os-miR528	transgene	[68]
***Virgaviridae***, Cucumber green mottle mosaic virus	miR156a, miR164, miR171a	transient transgene	[69,70]
***Tombusviridae***, *Tomato bushy stunt virus*	miR390a	transient	[71]
***Tombusviridae***, *Tomato bushy stunt virus*	miR171a	transient	[72]
***Alphaflexiviridae***, Potato virus X	miR159a, miR167b, miR171a	transgene	[57]
***Alphaflexiviridae***, Cymbidium mosaic virus	os-miR528	transgene	[68]
***Secoviridae,*** Grapevine fanleaf virus	miR319a	transient	[73]
***Spinareoviridae*,** dsRNA virus, Rice black streaked dwarf virus	zea-miR159a	transgene	[74]
***Spinareoviridae*,** dsRNA virus, Rice black streaked dwarf virus	os-miR528	transgene	[75]
***Phenuiviridae***, minus-RNA virus, Rice stripe virus	os-miR528	transgene	[75]
***Phenuiviridae***, minus-RNA virus, Rice stripe virus	os-miR528	transgene	[76]
***Tospoviridae***, minus-RNA virus, Watermelon silver mottle virus	miR159a	transgene	[77]
***Tospoviridae***, minus-RNA virus, Tomato spotted wilt virus	miR159a	transient, transgene	[78]
***Tospoviridae***, minus-RNA virus, Tomato spotted wilt virus	miR390a	transient, transgene	[31,79]
***Geminiviridae***, DNA virus, Tomato leaf curl New Delhi virus	miR159a, miR168a	transgene	[80]
***Geminiviridae***, DNA virus, Cotton leaf curl Burewala virus	miR169a	transgene	[81]
***Geminiviridae***, DNA virus, Wheat dwarf virus	hvu-miR171	transgene	[82]
***Geminiviridae***, DNA virus, Jatropha leaf curl Gujarat virus	miR159a	transgene	[83]
***Geminiviridae***, DNA virus, Tomato yellow leaf curl virus	miR159a	transgene	[84]
***Geminiviridae***, DNA virus, Tomato leaf curl virus	miR319a	transgene	[85]
***Geminiviridae***, DNA virus, Tomato yellow leaf curl virus	miR171 and miR164	transgene	[86]
***Geminiviridae***, DNA virus, Tomato yellow leaf curl virus	miR159a	transient	[87]
**Viroids,** Potato spindle tuber viroid	miR390a	transient	[88]
**Viroids,** plant gene VirP1	miR159a	transgene	[58]

* For novel standardized binomial names of the virus species, see https://www.ncbi.nlm.nih.gov/Taxonomy/, accessed on 16 November 2024.

Experiments with Tobacco etch virus (TEV) targeted by miR319a-based amiRNA in transgenic *N. tabacum* plants have also revealed significant inhibition of virus replication, and amiRNAs targeting the NIb and CP genes displayed a higher silencing efficiency than those targeting CI and NIa genes [59]. However, the authors provided no explanation for the observed phenomenon of the more efficient amiRNA constructs against NIb and CP genes [59]. An efficient artificial miRNA strategy has been developed against Wheat streak mosaic virus in transgenic *T. aestivum* (wheat). This strategy is based on incorporating five amiRNAs, which target 5′ UTR, P1, Hc-Pro, P3, and PIPO genes and are located within a single polycistronic amiRNA precursor. This polycistronic construct (FGmiR395) includes five different pre-miRNA hairpins corresponding to the mentioned genes and independently processed in transgenic plants from a single pri-miRNA [61]. Studies on Wheat yellow mosaic virus have shown that wheat transgenes expressing amiRNAs based on os-miR528 and targeting the NIb-coding genome region were highly resistant to virus infection [62] (Table 1).

In a recent study, *N. benthamiana* plants were transformed with amiRNA constructs based on miR159a, designed to target the CP and NIb genes of the potyvirus Plum pox virus (PPV) (Table 1). The obtained lines were highly resistant to PPV infection. Out of 7 lines with amiRNA against the NIb gene, four lines showed 100% resistant plants, two lines showed 40% resistant plants, and one line had all plants systemically infected. For amiRNA targeting CP gene, 9 lines resulted in 100% resistant plants, while two lines displayed 20% and 80% resistance. In contrast, all 7 lines with amiRNA constructs targeting the 3′-UTR were 100% susceptible to PPV infection [63]. Moreover, these experiments have shown that amiRNA directed against PPV genomic RNA is more efficient than amiRNA targeting its complementary strand [63]. It was shown recently that miR390a-based amiRNA can induce significant resistance to Zucchini yellow mosaic virus in transient experiments with agroinoculated *Cucurbita pepo* cotyledons [64]. Several constructs expressing amiRNAs, which are based on at-miR390a and targeting the Hc-Pro-coding region, were moderately resistant to virus infection. Agroinoculated cotyledons showed more than 40% protection [64].

#### 2.1.2. Family Bromoviridae

In early studies of amiRNAs against Cucumber mosaic virus (CMV, family Bromoviridae), it was shown that expression of amiRNAs targeting sequences encoding the silencing suppressor 2b of CMV by miR171a-based amiRNA in transgenic *N. tabacum* [65] and the 3′ untranslated region (3′-UTR) of the virus genome by miR159a-based amiRNA in transgenic *A. thaliana* and *N. tabacum* [66] can efficiently confer on transgenic plants effective resistance to CMV infection. Interestingly, transgenic plant lines that express ami-RNAs targeting the different regions in the 3′-UTR of CMV genomic RNA3 are not equally effective in conferring virus resistance because of extensive positional effects. Particularly, constructs that have the target site located in the tRNA-like sequence (TLS) region conferred no virus resistance. However, the constructs showing target recognition in the 5′ less-structured region beyond the TLS resulted in conferring different degrees of resistance to CMV infection [66].

Later, miR171a-based amiRNAs targeting polymerase (2a) and 2b genes as well as 3′-UTR have been shown to confer significant degrees of resistance to CMV infection in transgenic *Solanum lycopersicum* (tomato) plants. However, transgenic plants expressing amiRNA targeting the 2a and 2b viral genes display more efficient repression of CMV than amiRNA against the 3′-UTR because fruit weight in infected transgenic plants based on amiRNA against 2a/2b genes is 15% higher [67]. Importantly, grafting experiments, where the tomato plants were grafted onto a CMV-infected tomato rootstock, have revealed that the amiRNA signal against CMV cannot be transmitted over long distances through the vascular system [67] (Table 1).

#### 2.1.3. Family Virgaviridae

The first paper on the amiRNA against representative of genus *Tobamovirus* (family Virgaviridae) has described *Nicotiana benthamiana* transgenic lines expressing amiRNA based on the *O. sativa* miR528 as backbone and targeting RNA–polymerase gene of Odontoglossum ringspot virus [68]. However, only 16% of transgenic lines were partly resistant to virus infection.

More promising results have been obtained in the amiRNA studies on Cucumber green mottle mosaic virus (CGMMV, genus Tobamovirus). In a transient assay with agroinfiltrated *N. benthamiana*, Liang and co-authors have shown that amiRNAs based on miR156a, miR164, and miR171a efficiently inhibit multiplication of CGMMV when targeting coat protein, movement protein, and replicase genes, respectively [69]. Importantly, it has been found that virus resistance levels were varied for amiRNAs targeting different virus genes or the different regions of the same functional gene. For example, the resistance level was higher when the 5′ end of the Rep gene was targeted by amiRNA. However, the resistance level was lower when the middle segment of the MP gene was targeted [69]. In further study, stable transgenic *Cucumis melo* (cucumber) plants expressing polycistronic amiRNA against all above-mentioned CGMMV genes have been constructed, and these transgenic plants showed significant disease resistance against CGMMV [70] (Table 1).

#### 2.1.4. Family Tombusviridae

The *A. thaliana* miRNA390a backbone has been used to construct amiRNAs targeting the 5′-UTR of the Tomato bushy stunt virus (TBSV) genome. Transient expression of these amiRNAs in 4-to-6-week-old *N. benthamiana* plants via Agrobacterium-mediated infiltration showed a significant level of protective effect [71]. Construction and transient expression of miR171a-based amiRNA in *N. benthamiana* plants revealed that targeting coding regions of P33 and P19 proteins, similar to targeting UTR, inhibits TBSV multiplication (Table 1). Interestingly, it was also found that asymmetric bulges added in the duplex region of amiRNA could be used to improve amiRNA-mediated TBSV resistance, probably due to their ability to reduce binding of silencing suppressor P19 [72].

#### 2.1.5. Family Alphaflexiviridae

Significant resistance against the Potato virus X (PVX) has been demonstrated by targeting the silencing suppressor P25 (TGB1) gene using amiRNAs based on miR159a, miR167b, and miR171a backbones in transgenic *N. tabacum*. Additionally, transgenic *N. tabacum* showed highly effective resistance to both PVY and PVX through the expression of a dimeric amiRNA precursor [57]. In another study, authors generated *N. benthamiana* transgenic lines containing *O. sativa* miR528 as the backbone, which targets RNA-dependent RNA polymerase gene of Cymbidium mosaic virus (CymMV). These amiRNA transgenic lines express amiR528 against CymMV and confer a high percentage of resistance to virus infection (Table 1) [68].

#### 2.1.6. Family Secoviridae

The amiRNA-based strategy has also been reported in *Vitis vinifera* (grapevine). Two amiRNA precursors based on miR319a, targeting the coat protein gene of Grapevine fanleaf virus, have been constructed [73]. Transient expression of these pre-amiRNA constructs in grapevine somatic embryos after co-cultivation with A. tumefaciens has resulted in active processing of pre-amiRNAs by the plant machinery. However, the anti-viral activity of these amiRNAs has not been tested [73]. To confirm the biological functionality of the anti-CP gene amiRNA, a GUS-sensor assay was performed. The GUS–sensor constructs consisted of the GUS-encoding sequence fused to the amiRNA target sequence (21 nt size) in the 3′-UTR. To investigate amiRNA-induced gene silencing, co-transformation assays were conducted with a mix of Agrobacterium carrying a GUS–sensor construct and the corresponding the anti-CP gene amiRNA construct. Co-expression of the two constructs resulted in the significant silencing of the GUS gene by targeting the added virus-specific 21 nt sequence [73].

#### 2.1.7. Family Spinareoviridae

The sequence of the maize zea-miR159a precursor backbone has been used for the construction of amiRNA targeting the P6 silencing suppressor gene of the Rice black streaked dwarf virus (RBSDV). Disease caused by this dsRNA-containing virus brings significant damage to *Zea mays* (maize) production. The amiRNA plant expression vector was used for transforming maize by using the Agrobacterium-mediated method. Homozygous lines with high miRNA expression were selected and showed that the disease resistance of transgenic homozygous maize was much higher than that of wild-type plants [74].

Infection of rice with RBSDV also causes a significant loss of grain production. Three amiRNA precursor expression vectors that target the CP gene of RBSDV based on the osa-miR528 precursor have been used to construct transgenic *O. sativa* (rice). The transgenic plants were obtained by Agrobacterium-mediated transformation and shown to express amiRNAs successfully. Viral challenge assays revealed that these transgenic plants with the amiRNA-targeting 3′-UTR region of the CP gene demonstrated significant resistance (up to 54%) against RBSDV infection (Table 1) [75].

#### 2.1.8. Family Phenuiviridae

Rice stripe virus (RSV) is a minus-stranded RNA virus and represents a significant agricultural threat. Dimeric amiRNAs based on osa-miR528 and targeting CP genes of RSV and RBSDV (see above) were used for constructing transgenic rice plants. These transgenes showed obvious resistance to both viruses [75]. Significant resistance against RSV has also been demonstrated in transgenic rice plants expressing osa-miR528-based amiRNA targeting the virus movement protein gene (Table 1) [76].

#### 2.1.9. Family Tospoviridae

It was shown that several designed amiRNAs based on miR159a as the backbone and targeting the replicase (L) gene confer moderate resistance against Watermelon silver mottle tospovirus in transgenic *N. benthamiana* plants. Moreover, the multimeric construct including the two amiRNAs targeting the L gene provides complete resistance [77]. The miR159a backbone was also used to construct amiRNAs targeting nucleocapsid (N) and silencing suppressor (NSs) genes of Tomato spotted leaf virus (TSWV). Transient expression of these amiRNAs in *N. benthamiana* demonstrated that plants expressing N-specific amiRNAs showed high resistance against TSWV. However, plants expressing NSs-specific amiRNAs were symptomatic and accumulated high levels of TSWV. Similar findings were obtained in stably transformed *N. tabacum* plants [78]. Additionally, it has been shown in independent studies with TSWV that expression of the miR390a-based amiRNAs targeting replicase and movement protein genes resulted in significant virus resistance of *N. benthamiana* plants and *S. lycopersicum* (Table 1) [31,79]. Taken together, these results indicate that only a subset of amiRNAs targeting TSWV segments L (polymerase gene) or M (movement protein gene) were highly active and blocked TSWV systemic infection in *N. benthamiana*, while none of the amiRNAs against fragment S (NSs gene) were effective [79].

#### 2.1.10. Family Geminiviridae

Many studies have shown that amiRNA-mediated technology is efficient not only against RNA plant viruses but also against DNA viruses. Initially, these studies were performed with the members of genus Begomovirus, family Geminiviridae. Particularly, two amiRNA precursors based on tomato miR159a and miR168a and targeting the overlapping region of the coat protein gene (AV1) and AV2 gene of the Tomato leaf curl New Delhi virus have been constructed. It was demonstrated that transgenic *S. lycopersicum* plants expressing amiRNA were highly tolerant to virus infection [80]. Similarly, the amiRNA construct based on the miR169a sequence and targeting the V2 gene of the Cotton leaf curl Burewala virus showed good virus resistance in transgenic *N. benthamiana* [81].

In studies of Wheat dwarf virus (WDV, genus *Mastrevirus*, family Geminiviridae), amiRNA based on the *Hordeum vulgare* (barley) hvu-miR171 precursor backbone was designed to target conservative sequence elements of the WDV replication-related genes. A polycistronic amiRNA precursor construct was built to express three amiRNAs simultaneously. This construct was transformed into barley under the control of a constitutive promoter, resulting in significant resistance to insect-mediated WDV infection (Table 1) [82].

The design and expression of amiRNAs in transgenic tobacco plants confirmed that the amiRNA constructs targeting replication-related and silencing suppressor genes of begomovirus Jatropha leaf curl Gujarat virus conferred virus resistance. However, the amiRNA transgenics targeting the coat protein gene were only partly tolerant to virus infection [83]. Independent studies with begomovirus Tomato yellow leaf curl virus (TYLCV) have also shown that amiRNA based on miR159a and targeting replication-related gene AC1 induced a substantial decrease in virus replication in transgenic tomato plants [84]. Likewise, an amiRNA was developed on the backbone sequence of Arabidopsis miRNA319a and targeting the RNA sequence coding for the ATP/GTP binding domain of the AC1 gene of the begomovirus Tomato leaf curl virus (TLCV). Transgenic tomato lines over-expressing this amiRNA, when challenged with TLCV, showed reduced disease symptoms and high percentage resistance ranging between ~40 and 80%. The yield of transgenic plants was significantly higher upon virus infection as compared to the non-transgenic plants [85]. Interestingly, the multimeric amiRNA, targeting seven genes of TYLCV and based on Arabidopsis miR171 and miR164 precursors as the basic backbones, are able to confer higher virus resistance in transgenic tomato plants when amiRNAs of different gene specificity are separated by plant-specific introns (Table 1) [86]. TYLCV was also used as a model system for demonstrating an alternative delivery method of the plant vector DNA possessing amiRNA constructs. Recombinant plasmid DNAs of amiRNA constructs based on miR159a and targeting AV1 and AV2 genes were loaded onto nontoxic and degradable layered double hydroxide (LDH) clay nanosheets. These LDH nanosheets were sprayed onto plant leaves, and the infectivity of TYLCV in *N. benthamiana* and *S. lycopersicum* plants harboring amiRNA was significantly suppressed after foliar spraying [87].

#### 2.1.11. Viroids

Viroids are the smallest non-cellular pathogens and represent the non-coding, infectious circular RNAs that are known to be highly structured and able to replicate autonomously in the host plants. Although they do not encode any peptides, viroids induce visible symptoms in susceptible host plants and represent an obvious threat for agriculture [89,90,91]. Initially, five amiRNAs based on miR390a and targeting RNA chains of both polarities in Potato spindle tuber viroid (PSTVd) have been shown to cause significant delay of viroid accumulation in agroinoculated *N. benthamiana* (Table 1) [88].

However, the use of amiRNA targeting the viroid RNA sequences may cause deleterious effects in transgenic plants. Particularly, expression of some PSTVd sequences from the virulence-modulating region as amiRNAs resulted in phenotypes reminiscent of viroid-infected potato plants. Furthermore, the severity of the phenotypes displayed was correlated with the level of amiRNA accumulation. It was found that these deleterious viroid-targeting amiRNAs may also target important plant genes such as the transcription factor-encoding gene StTCP23 from *S. tuberosum* [92], the soluble inorganic pyrophosphatase gene from *N. tabacum* [93], and the STEROL GLYCOSYLTRANSFERASE 1 gene from tomato [94]. Alternatively, it was recently proposed to use amiRNA for targeting plant genes, as they are important for viroid replication. Transgenic plants of *S. tuberosum* were obtained to target the VirP1 gene required for the nucleo-cytoplasmic transport of PSTVd by Agrobacterium tumefaciens-mediated transformation. These transgenic plants could express multimeric amiRNAs based on miR159 and target the VirP1 gene, TGB1 of PVX, and HcPro of PVY. Viral (viroid) challenge assays revealed that these transgenic plants demonstrated resistance against PVX, PVY, and PSTVd coinfection simultaneously [58].

### 2.2. Plant Virus Vectors for Expression of amiRNA

It has been shown that plant viruses are potentially important for biotechnology not only as pathogens but also as promising vectors for the expression of amiRNAs. In animal systems, it has been reported that (i) a recombinant vector Snakehead rhabdovirus expressing amiRNA targeting genome sequences of Spring viremia carp virus (SVCV) protects zebrafish (*Danio rerio*), resulting in significantly higher survival rates than fish infected with SVCV alone [95]; (ii) a recent study carried out with the adeno-associated virus 8 (AAV8) vector-mediated gene delivery in mouse model system has shown that the expression of anti-Hepatitis B virus amiRNA significantly inhibited the replication of different HBV genotypes, and the expression of HBV antigens in serum and liver tissue was strongly suppressed after a single intravenous vector injection [96].

In plant models, both DNA- and RNA-containing virus vectors have also been used for the expression of amiRNAs. A recent paper has reported triggering widespread silencing in a non-transgenic manner by using the Potato virus X vector for transient expression of miR390a-based amiRNAs targeting endogenous plant genes in *N. benthamiana* [97]. However, an earlier study has shown a direct comparison of DNA virus vector (Tomato mottle begomovirus—ToMoV) and RNA virus vector (Tobacco mosaic virus—TMV), both expressing amiRNA based on miR319a and targeting the phytoene desaturase (PDS) mRNA of *N*. *benthamiana*. These results revealed that the ~21 nt RNAs expressed from the TMV vector did not have the precise cleavages as predicted for the miRNA pathway. By contrast, the additional analyses showed that infection by the ToMoV vector gave specific production of 21 nt amiRNAs, indicating precise cleavages in the miRNA sequence, and showed reduced NbPDS transcript accumulation levels [98]. Other plant DNA viruses also showed promising results in attempts to express amiRNA. It was found that a modified Cabbage leaf-curl geminivirus vector can be used to express amiRNAs in plants and was effective to silence the expression of endogenous genes, including PDS, Su, CLA1, and SGT1, in *N*. *benthamiana* [99]. In addition, the use of viral satellite DNA associated with the Tomato yellow leaf curl China virus as a vector has shown that the miR390a backbone-based amiRNAs are highly overexpressed from this vector and specifically silence the targeted PDS gene in *N. benthamiana* [100].

### 2.3. Expression of amiRNA Against Non-Viral Plant Pathogens

Plants counteract various stresses during their growth and development, including pest infestation. Exploring the relationship between miRNAs and plant biotic stress responses expedites research projects to develop new resistant plant varieties. Apart from being used in engineering the plant antiviral systems, designing and expressing amiRNA has been commonly regarded as a tool to create plant resistance against other pathogens such as bacteria, fungi, and insects. Currently, most research papers are related to the application of amiRNA in developing resistance to insect pests in plants, and much less studies investigate amiRNA as a tool to inhibit plant infections by bacteria and fungi [see reviews [16,27,28,30,32,34,35,101,102,103]].

Recently, promising results have been obtained in studies of the amiRNA-mediated silencing of endogenous plant defensins in *A. thaliana* [51]. The authors constructed transgenic plants expressing the multimeric miR319a-based amiRNA to silence a group of AtPDF genes. Unexpectedly, the amiRNA-expressing plants were more resistant than wild-type plants to infection by different pathogens, such as the fungus *Botrytis cinerea*, the oomycete *Hyaloperonospora arabidopsidis*, and the bacteria *Pseudomonas syringae*. Evidently, further studies are necessary to clarify the underlying mechanisms responsible for this phenomenon [51]. Another unexpected result has been found in the research of engineered resistance against Wheat yellow mosaic bymovirus (WYMV) in wheat transgenes expressing amiRNAs based on osa-miR528 and targeting the NIb-coding genome region [62]. Laboratory and field tests showed that transgenic wheat lines expressing amiRNA were highly resistant to WYMV infection and, moreover, revealed a broad-spectrum disease resistance to Chinese wheat mosaic virus, Barley stripe mosaic virus, and rust fungus *Puccinia striiformis* infections. The authors suggest that these results can be explained by the occasional activation of host immunity by amiRNA-mediated regulation of plant defense-related genes via ROS signaling [62].

Modified Arabidopsis pre-miR164b was used as a backbone for amiRNA targeting the Avr3a gene from the oomycete *Phytophthora infestans* (potato late blight agent). Five different amiRNA constructs targeting several regions of the P. infestans Avr3a cistron were expressed in transgenic potato. Four transgenic lines were moderately resistant to *P. infestans*, and the level of Avr3a transcript was reduced [104].

The rice gene xa13 is a recessive resistance allele of a member of the NODULIN3 gene family, which represents a susceptibility gene for bacteria *Xanthomonas oryzae*, the causal agent of bacterial blight [105]. It has been suggested that the conversion of bacterial blight resistance mediated by the recessive xa13 gene into a dominant plant property is important for rice breeding programs. This conversion was achieved by silencing the dominant allele Xa13 in transgenic rice using the expression of the corresponding amiRNA under tissue-specific promoters to ensure that Xa13 functioned normally during pollen development [105]. In another study, it was reported that the CmMLO17 gene in *Chrysanthemum morifolium* was upregulated after infection by the ascomycete fungus *Alternaria alternata*. Silencing of the CmMLO17 gene by amiRNA based on the miR319a backbone resulted in reduced susceptibility of transgenic chrysanthemum to *A. alternata* infection. The authors speculated that amiRNA-expressing transgenic plants had a faster and stronger defense response that was mediated by the ABA and Ca^2+^ signaling pathways, resulting in reduced susceptibility of chrysanthemum to A. alternata infection [106].

As previously mentioned, the application of amiRNA for developing resistance to insect pests in plants is a rapidly progressing area of research. Generally, amiRNAs may induce insect-specific gene disorders in feeding pests by regulating the expression of their target genes and, thereby, causing abnormal development, death, or reduced drug resistance [28,34,102]. For expression in plants, two kinds of amiRNAs can be used. First, amiRNAs based on plant pre-miRNA backbones produce mature anti-insect miRNA by the endogenous plant DICER system and, then, are subjected to inter-kingdom small RNA transfer into insect organisms, thereby conferring the plant with insect resistance. In a pioneering work, the Arabidopsis pre-miR171a was utilized as a backbone to create amiRNA that targets the acetylcholinesterase 2 gene (*MpAChE2*) in the aphid *Myzus persicae.* This led to reduced expression levels of the *MpAChE2* transcript in aphids feeding on amiRNA transgenic tobacco plants. Insect challenge assays showed that most of the transgenic plant lines gained aphid resistance [107]. The plant pre-miRNA backbone (sly-miR-159) was used to generate the construct for the expression of amiRNA (amiR-24), which targets the chitinase gene of *Helicoverpa armigera* as a transgene in *N. tabacum*. The transgenic plant line with the high level of expression of mature amiR-24 showed a significant level of insect death upon larval feeding [108]. Silencing of the insect *HaAce1* gene by amiRNA based on plant miR164b and expressed in transgenic tobacco disrupts the growth and development of *H. armigera*. Increased larval mortality of 25% and adult deformity of 20% were observed in transgenic plants in comparison with control tobacco [109]. It was also reported that the transgene-expressed amiRNA, based on the rice osa-miR162a backbone and targeting the *NlTOR* (Target of rapamycin) gene, regulates the reproduction process of brown planthopper (*Nilaparvata lugens*). Transgenic overexpression of this amiRNA conferred rice resistance to planthopper without detectable developmental penalty of plants [110]. Additionally, insect-specific microRNA, miR-14, of *Chilo suppressalis*, which was predicted to target Spook (Spo) and Ecdysone receptor (EcR) in the ecdysone signaling network, was used to produce amiRNA based on rice osa-miR528. Feeding bioassays using both T_0_ and T_1_ generations of transgenic rice indicated that at least one line showed high resistance to striped stem borer [111]. 

In the second approach, amiRNAs based on insect pre-miRNA and targeting vital insect genes are produced in transgenic plants, where these molecules remain largely unprocessed. However, these amiRNAs are able to transfer into insect organisms. Then, recognition by the insect dicing system occurs that suppresses pest infestation [28]. A series of insect pre-microRNAs (*Diabrotica virgifera* miR279b and miR1) were modified to produce amiRNAs, targeting the insect vital acetylcholinesterase 2 gene, and expressed in transgenic *Nicotiana benthamiana* plants. *H.* armigera feeding on leaves from these plants had increased mortality, developmental abnormalities, and delayed growth rates [112]. Transgenic rice plant lines expressing amiRNA based on miR260 of C. suppressalis (striped stem borer) and targeting cytochrome P450 enzyme, catalyzing the C_22_-hydroxylation of 2, 22-dideoxy-3-dehydroecdysone, showed a high level of larval mortality at 35 days after insect feeding [113]. Some other papers on transgenic rice plants, expressing amiRNA that target several genes of *C. suppressalis*, have also shown significant growth inhibition for feeding larvae [114,115]. 

### 2.4. Expression of amiRNA for Regulating Plant Development, Yield Improvement, and Abiotic Stress Tolerance

There exist strategies for plants to respond and adjust to abiotic stressful circumstances. It is commonly accepted that major constraints to crop production are water shortage, salinity, low temperature, high temperature, heavy metal exposure, and high light intensity. Throughout their evolution, plants have improved and developed mechanisms in which miRNAs play a critical role in promoting abiotic stress tolerance [27,34,116]. Specifically, amiRNA technologies resulted in creating transgenic plant lines that can accumulate more zinc and cadmium and avoid shoot toxicity symptoms [117]. Moreover, there are also reports on the use of amiRNAs targeting stress-responsive plant genes that result in higher tolerance to drought stress in *S. tuberosum* (potato) plants [118,119].

In a recent review, different approaches for the improvement of vegetable, fruit, and flower horticultural crops by amiRNA techniques have been summarized. The improved traits include, particularly, reduced enzymatic browning of potato tubers, early ripening in tomato, increased sucrose levels in potato tubers, increased flowering duration in chrysanthemum, smaller fruits in tomato, and changes in flower size and color [34].

## 3. Potential Biotechnological Impact of Artificial Plant miPEPs

Many studies have shown that some plant pri-miRNAs encode regulatory peptides, referred to as miPEPs [38,40,120]. The exogenous administration of the chemically synthesized miPEPs specifically increases their cognate pri-miRNA transcription and miRNA accumulation. Importantly, it is generally agreed upon that external application of artificial synthetic miPEPs, via irrigation or leaf spraying of plants, is a promising avenue for improving agronomic traits [38,39,40,41,120,121,122,123,124,125,126,127,128]. Currently, many hundreds of unknown miPEPs can be predicted through several bioinformatic tools to expedite their finding and studies in fundamental and applied areas [129]. 

Many miPEPs may help plants resist biotic/abiotic stresses and grow faster. This leads to better root growth, early flowering, increased stem height, and, as a result, helps yield enhancement [38,40,121,126,127,128]. Interestingly, recent studies have revealed one of the modes to regulate two potentially agronomically important miPEPs (At-miPEP858a and At-miPEP408). It was found that the corresponding pri-miRNAs are regulated by light via the shoot-to-root mobile transcription factor HY5 in *A. thaliana* [130,131]. The application of these synthetic peptides to *A. thaliana* plants results in modulating the expression of their pri-miRNA through the involvement of auxin response elements leading to the regulation of growth, particularly to early bolting and a significant increase in the plant’s height [132,133].

The total root mass can be increased by chemically synthesized peptides At-miPEP166g and At-miPEP397a, whereas Gm-miPEP172c promotes nodulation with the increase in nodule numbers in *Glycine max* (soybean) [121,134]. Root modifications have been found after the external application of synthetic At-miPEP165a, Bo-miPEP156a, and Mt-miPEP171b in the genera *Arabidopsis*, *Brassica*, and *Medicago*, respectively. Similarly, Vvi-miPEP171d1 positively regulates adventitious root formation in *V. vinifera* (grapevine) [121,135,136]. Moreover, it has been found that synthetic peptides Bo-miPEP397a and Bv-miPEP164b are able to increase total plant size and the foliar surface in *Brassica oleraceae* and *Barbarea vulgaris* [121]. However, synthetic At-miPEP408 increases the sensitivity of seedlings toward low sulfur and arsenite As(III) stresses after addition to the growth media [137].

When applied to *V. vinifera* plantlets, synthetic Vvi-miPEP172b and Vvi-miPEP3635b increase cold tolerance [138]. The application of synthetic Os-miPEP156e reduces the inhibition of rice seedling growth after Cd stress [139]. Likewise, the synthetic rice peptides Os-miPEP172b, Os-miPEP528, Os-miPEP396c, Os-miPEP171c, and Os-miPEP166b increase resistance to Cd stress [139].

It has been shown that the exogenous application of At-miPEP169c, At-miPEP169h, and At-miPEP396b can stimulate significant plant resistance to the necrotrophic fungus *Botrytis cinerea*. Spraying bean leaves with *Phaseolus vulgaris* miPEPs Pv-miPEP169h, Pv-miPEP169k, and Pv-miPEP169p makes them more resistant to this necrotrophic fungus [140]. According to Ormancey and co-authors, four strawberry peptides, namely Fv-miPEP169h, Fv-miPEP169l, Fv-miPEP396a, and Fv-miPEP396f, significantly enhance plant fruit resistance following leaf inoculation with *B. cinerea* spores [140]. The exogenous application of tomato Sl-miPEP169d also confers the protective effect on the *S. lycopersicum* against the bacteria *Pseudomonas syringae* and *Xanthomonas* sp., as well as the fungus *Alternaria solani* [140].

Recent research studies have revealed the potential of externally applied miPEPs to alter secondary metabolic pathways and boost the production of secondary metabolites, notably anthocyanins, which are essential for grape quality and possess bioactive properties. It was demonstrated that Vvi-miPEP164c stimulates anthocyanin synthesis by the inhibition of the proanthocyanidin pathway, as both pathways compete for the same substrate [141,142].

Very recently, a novel method based on the exogenous application of designed synthetic peptides (cPEPs), recognizing their own coding regions in the 5′-terminal areas of mRNAs and enhancing their translation, was also shown to have good perspectives in plant agriculture for positive regulation of desired genes [143,144].

## 4. Conclusions

The regulatory capabilities of amiRNAs and miPEPs can be harnessed to develop safer, more sustainable agricultural practices that will enhance crop resilience. However, it is crucial to comprehend the entire mechanisms of miPEP and amiRNA biogenesis and mode of action, from transcription-related aspects to molecular ways of targeting specific genes. Driven by the increasing demand for high-end agricultural products, the amiRNA and miPEPs technologies can provide effective means to precisely modify gene expression associated with desired agronomic traits. However, very little research has been conducted on the utilization of these technologies for modifying quality traits such as improving nutrition composition, flavor, aroma, color, and edibility in horticultural crops. The regulation of expression of ripening-specific genes through amiRNA and miPEPs technologies can also be utilized to enhance the post-harvest shelf life of vegetables and fruits. Evidently, more focused studies considering plant amiRNA and miPEPs will provide more insightful findings and mechanistic evidence concerning plant growth, plant resistance to abiotic factors, and plant–pathogen interaction.

In summary, significant progress is clearly being made in elucidating the significance of amiRNAs in conferring plant immunity against viroids, insects, viral, and some microbe pathogens. However, the comprehension of the interaction between amiRNAs and fungal/bacterial pathogens remains limited. Future research should further elucidate the role of amiRNAs and identify novel amiRNAs against microbe pathogens. Additionally, it is imperative to explore the functions of key components within the plant signaling pathways to enhance the application of the plant gene-targeting amiRNAs in improving broad plant disease resistance.

The primary goal of this review is to show the readers that our primary objective of researching amiRNAs and plant peptides (miPEPs) is to foster agricultural advancement. Notably, the application of artificially produced derivatives of miRNA genes to plants will have increasing success in effectively regulating growth, development, and biotic/abiotic stress responses.
